# Miscellaneous Notices

**Published:** 1851-07-01

**Authors:** 


### Miscellaneous Notices.

*Practical Remarks on some Exhausting Diseases.* By Sir JAMES EYRE, M.D., &c. 2nd edition. London: J. Churchill. 1851.

WE are glad to see a second edition of this work. The profession owes a debt of gratitude to the author for having directed attention to the exhibition of the oxide of silver in certain exhausting diseases. Sir J. Eyre and other eminent practitioners have fully tested the efficacy of this remedial agent, and speak highly of it. The author has had, since the first edition of his work appeared, frequent opportunities of witnessing the beneficial effect of the oxide of silver in certain hæmorrhagic conditions of the system peculiar to the department of practice in which he is specially engaged, and he has great faith in the remedy if perseveringly and judiciously administered. Sir J. Eyre writes like a man fully impressed with the truth of what he is stating. We believe him to be incapable of printing what he does not know to be the fact; and this, of course, gives additional value to anything that proceeds from his pen. The work does him credit, and we warmly recommend it to our readers.

---

*Cholera in the West Yorkshire Lunatic Asylum.* By T. G. WRIGHT, M.D. London. 1850.

THIS work is devoted to the consideration of the origin and progress of cholera in the West York Lunatic Asylum during the autumn of 1849. It is an able production, and must have caused the author considerable labour. The work is full of statistical data and tabular statements, evidently drawn up with great care.

---

*Thoughts on Insanity and its Causes, and on the Management of the Insane.* By a MECHANIC. London: C. Gilpin, Bishopsgate-street. 1851.

A VERY sensible and well-written little work. The author is, however, disposed to take too spiritual a view of insanity; but his views are so modestly put forward that they are entitled to every respect and attention. We are glad to see thinking, reading, and observing men in the author's rank of life giving the profession the benefit of their opinions. In the next number of our Journal we purpose extracting some passages from the pamphlet.

---

*Autobiography of the Rev. William Walford.* Edited by JOHN STOUGHTON. London: Jackson & Walford. 1851.

THIS is a most valuable piece of biography, particularly so to the psychologist, and all interested in the workings of a mind under the influence of disease. We fully intended to have quoted largely from the volume, but are reluctantly compelled to defer this pleasure until another occasion.

---

WE will publish, in our next number, Analyses of the Foreign Journals, List of Books, and Extracts from the Reports of British Asylums. We have again to complain of the postage charged for parcels of books and pamphlets coming fro mAmerica. We paid, for the last number of the *American Journal of Insanity*, and the *Report of the State Lunatic Asylum, New York*, twelve and sixpence postage! This should not be. Parcels of books, if sent through the post, should be open at each end; but the better plan is to forward all books &c. through a respectable London agent, addressed to the Editor.

Mr. Baily's work, on the "Theory of Reasoning," and the new edition of Dr. Carpenter's "Physiology," will be fully reviewed in our October number, with other books forwarded to us.

The Editor's account of a recent visit to the Royal Hospital, Charenton, and his *Essay on Prison Discipline*, read at the Medical Society of London, are unavoidably postponed, in consequence of press of matter, until our next number.

